# The intrinsic defect structure of exfoliated MoS_2_ single layers revealed by Scanning Tunneling Microscopy

**DOI:** 10.1038/srep29726

**Published:** 2016-07-22

**Authors:** Péter Vancsó, Gábor Zsolt Magda, János Pető, Ji-Young Noh, Yong-Sung Kim, Chanyong Hwang, László P. Biró, Levente Tapasztó

**Affiliations:** 1 Centre for Energy Research, Institute of Technical Physics and Materials Science, 2D Nanoelectronics „Lendület” Research Group, Budapest, Hungary; 2 Korea Research Institute of Standards and Science, Center for Nanometrology, Daejeon, South Korea; 3 Centre for Energy Research, Institute of Technical Physics and Materials Science, Nanotechnology Department, Budapest, Hungary

## Abstract

MoS_2_ single layers have recently emerged as strong competitors of graphene in electronic and optoelectronic device applications due to their intrinsic direct bandgap. However, transport measurements reveal the crucial role of defect-induced electronic states, pointing out the fundamental importance of characterizing their intrinsic defect structure. Transmission Electron Microscopy (TEM) is able to image atomic scale defects in MoS_2_ single layers, but the imaged defect structure is far from the one probed in the electronic devices, as the defect density and distribution are substantially altered during the TEM imaging. Here, we report that under special imaging conditions, STM measurements can fully resolve the native atomic scale defect structure of MoS_2_ single layers. Our STM investigations clearly resolve a high intrinsic concentration of individual sulfur atom vacancies, and experimentally identify the nature of the defect induced electronic mid-gap states, by combining topographic STM images with *ab intio* calculations. Experimental data on the intrinsic defect structure and the associated defect-bound electronic states that can be directly used for the interpretation of transport measurements are essential to fully understand the operation, reliability and performance limitations of realistic electronic devices based on MoS_2_ single layers.

Beyond graphene, alternative two-dimensional materials, such as transition-metal dichalcogenides (TMDCs), are in the focus of scientific attention due to their intrinsic direct bandgap and various intriguing properties^1^,^2^ Single-layer molybdenum disulfide (MoS_2_), is one of the most intensively studied materials from the TMDC family. Compared to the bulk and few layer MoS_2_, the monolayer form is a direct band gap semiconductor^3^,^4^ that makes it a good candidate for transistor^5^,^6^, photodetector^7^ and optoelectronic^1^,^8^,^9^ device applications. However, a clear drawback of the MoS_2_ single layers is the much lower charge carrier mobility as compared to graphene. One of the main culprits for the reduced mobility is a high concentration of intrinsic structural defects as indicated by electrical transport measurements^10^,^11^. The critical role of defects in the electronic behavior of MoS_2_ single layer devices is clearly demonstrated by Qiu *et al*.^10^ who reported a novel defect-mediated transport mechanism where the transport is dominated by hopping via defect induced localized states. These findings clearly evidence that not only the type and concentration, but also the spatial distribution of the native structural defects of MoS_2_ single layers are critical for the correct interpretation of their electronic properties. Apart from the electronic device applications defects play an increasingly important role in the two-dimensional limit of the materials, also influencing their mechanical, optical or catalytic properties^12^. Therefore, experimentally characterizing the intrinsic concentration, distribution, as well as the atomic and electronic structure of the native defects of MoS_2_ monolayers is of key importance for the development of the field.

TEM investigations have been successfully used to image atomic defects in MoS_2_ single layers, revealing a high concentration of point defects, identified as sulfur vacancies^13^. Other kind of defects, such as interstitial defects^14^, dopant impurities^15^, dislocations and grain boundaries^16^,^17^,^18^ have also been observed. However, the TEM technique has some clear limitations in characterizing the intrinsic defect structure of TMDC single layers. First, it does not only image, but also induces novel point defects in the MoS_2_ sheet^19^; therefore, determining the native defect concentration is not straightforward. Furthermore, it also modifies the intrinsic spatial distribution of defects by providing enough energy to activate their migration, even their coalescence into line defects^20^. Another drawback is that TEM does not provide experimental information on the electronic structure of the defects, essential for the interpretation of transport properties and device characteristics.

In principle, Scanning Tunneling Microscopy is an ideal tool to overcome the above mentioned limitations of the Transmission Electron Microscopy. Due to the low energy of the tunneling electrons, no modification of the intrinsic defect structure is expected; therefore, the native defect density and distribution can be directly determined from the STM images. Furthermore, the information gained by the STM, together with theoretical calculations can also give insight into the electronic structure of the defects.

Although STM has a long history in the investigation of surface defects of bulk TMDCs^21^ including MoS_2_ 22,23,24,25,26,27, resolving the structure of atomic scale native point defects in MoS_2_ single and few-layers turned out to be challenging^11^,^28^,^29^. Previous STM investigations mainly revealed nanometer scale modifications of the local density of states extending over several atomic sites, but no direct imaging of atomic scale structural discontinuities such as a single S atom vacancy could be achieved. This might be related to the fact that the defect states lie inside a relatively wide (1.5–2 eV) band gap. The inability of the STM to fully resolve atomic scale point defects in MoS_2_ also leads to a discrepancy between the defect density apparent from the STM images^11^,^27^,^28^,^29^ and those experimentally estimated based on TEM investigations^10^,^13^.

Here, we report the successful atomic resolution STM investigation of large area exfoliated MoS_2_ single layers fully revealing their intrinsic atomic scale defect structure, as well as providing information on the electronic properties of the native structural defects.

## Experimental

We have mechanically exfoliated synthetic crystals of bulk MoS_2_ of high structural quality (2DSemiconductors) to investigate the lower limit of the native defect density from the available samples. Investigating high quality exfoliated samples instead of CVD grown layers can provide information on the intrinsic limitations and ultimate device performance of MoS_2_ single layers. We have employed a novel mechanical exfoliation technique developed by us^30^, providing single layer MoS_2_ flakes with typical lateral size of order of hundreds of microns on atomically flat Au (111) surfaces. The single layer nature of the exfoliated MoS_2_ flakes has been confirmed by Raman spectroscopy. The exfoliation occurs under mild sonication. To exclude the possibility that additional defects are introduced during the preparation step, we compared samples subjected to various sonication times, but did not observe significant change in their defect density. Large area flakes can be easily identified under an optical microscope enabling the guided landing of the STM tip on MoS_2_ single layers. We have performed the STM investigations at room temperature and under ambient as well as pure nitrogen atmosphere. Atomic resolution imaging could be easily achieved under these conditions. Although imaging the hexagonal lattice of the top layer of sulfur atoms can be routinely achieved, the clear observation of individual point defects turned out highly challenging. Under typically employed STM imaging condition for MoS_2_ samples (i.e. imaging outside of the bandgap, with |U _bias_| > 1 V) only nanometer scale modifications of the local density of states could be observed, similar to those previously reported in the literature^11^,^27^,^28^. We attribute these features to not completely resolved defect clusters. However, we found that the atomic structure of individual point defects of the MoS_2_ single layers can be clearly resolved when imaging at much lower bias voltages, of order of tens of meV. This implies that we are imaging within the band gap, which was nevertheless possible for atomically thin MoS_2_ single layer laying on gold substrate. We attribute the ability to image within the gap to the influence of the Au(111) substrate, which has been shown to induce a small but finite density of states also within the MoS_2_ band gap, conferring a weak metallic character to the MoS_2_ single layers deposited on Au(111)^31^,^32^. This is in good agreement with our experimental tunneling spectroscopy data (see Fig. S2 in the SI), revealing a finite density of states within the MoS_2_ bandgap.

However, even at low bias voltages, clearly resolving individual atomic point defects was not always possible (see SI for details). For the defects to clearly appear in low bias STM images the Fermi level of the imaged MoS_2_ layer has to be located very close to the energy of a defect state. In our experiments we could not directly control the position of the Fermi level; instead we exploited the intrinsic variation of the Fermi level position within the flakes^11^ to find sample areas with the right Fermi level position. However, the Fermi level position can also be easily controlled by gating the MoS_2_ sample within the STM^28^.

Our STM investigation also revealed, that even after long scanning times the number of defects and their spatial distribution remained unchanged in the scanning area, evidencing the truly noninvasive nature of the STM measurements, and enabling us to image the intrinsic defect structure of high quality exfoliated MoS_2_ single layer samples.

## Results and Discussion

**Figure 1:**
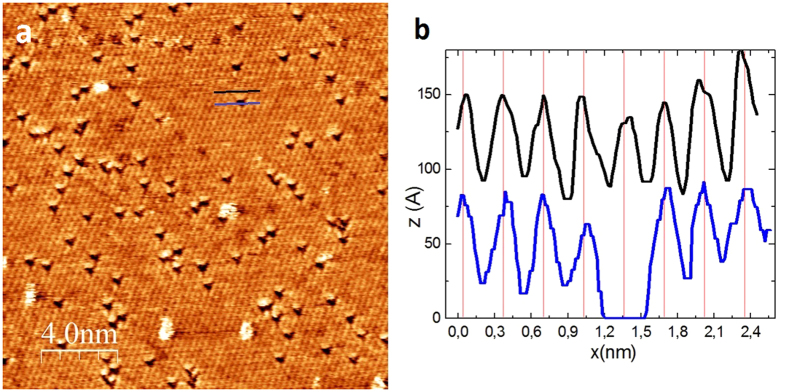
Atomic resolution STM images of native point defects in MoS_2_ single layers. (**a**) Atomic resolution STM image (*V*_*b*_ = 50 mV, *I*_*t*_ = 2 nA) clearly resolving individual point defects (dark triangles) present in a high (10^13^ cm^−2^) native concentration in mechanically exfoliated MoS_2_ single layers on Au (111) substrate. (**b**) Line cuts displaying the atomic corrugation along the lines marked in panel (*a*) and revealing that the imaged defects (dark triangles) are centered on the position of an empty sulfur lattice site, indicating their S atom vacancy nature.

Figure 1a shows a typical atomic resolution STM image (20 nm × 20 nm) after optimizing the imaging parameters to fully reveal the atomic scale defect structure of exfoliated MoS_2_ single-layers on Au(111) substrate. The basic pattern observed is a hexagonal lattice of 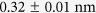 periodicity, corresponding to the atomic lattice of the top sulfur layer of the 2D MoS_2_ crystal. Larger bright features are contaminants (adsorbed molecules) that have been frequently moved (swept) during the scanning. By contrast, dark triangles are atomic scale discontinuities stable both in number and position during long scanning times. Line profiles (Fig. 1b) across the dark triangles reveal that they are centered on a lattice site of the top sulfur layer, except that no S atom is present, indicating their S vacancy nature. The experimentally observed positions of the defects (centered on S lattice sites) exclude several other defect types, such as interstitials, Mo vacancy or antisite defects (a Mo atom substituting a S_2_ column or vice versa). Furthermore, the observed clear dominance of sulfur vacancies is in agreement with the theoretical calculations concerning the formation energies of the different types of structural defects in single-layer MoS_2_. DFT formation energy calculations predict the prevalence of sulfur vacancies against other types of point defects like Mo vacancy, interstitial or antisite defects^13^,^14^,^33^,^34^.

Another important observation is that during the STM investigation we have not seen the introduction of novel point defects; therefore, we can directly determine the intrinsic defect concentration in high quality exfoliated MoS_2_ single layers. Based on STM images of several samples, we found that the distribution of the point defects is non-uniform across the sample surface, with their native concentration typically ranging from 5 × 10^12^ to 5 × 10^13^ cm^−2^ values. These values are comparable to the results of TEM investigations where a point defect concentration of the same order of magnitude has been estimated indirectly, through extrapolation^10^,^13^. However, this is a much larger defect density than apparent from pervious STM investigations^11^,^28^,^29^ of MoS_2_ single and few-layer samples. We attribute this to the fact that in contrast to previous STM measurements, we were able to fully resolve the individual atomic scale defects in MoS_2_ single layers.

**Figure 2:**
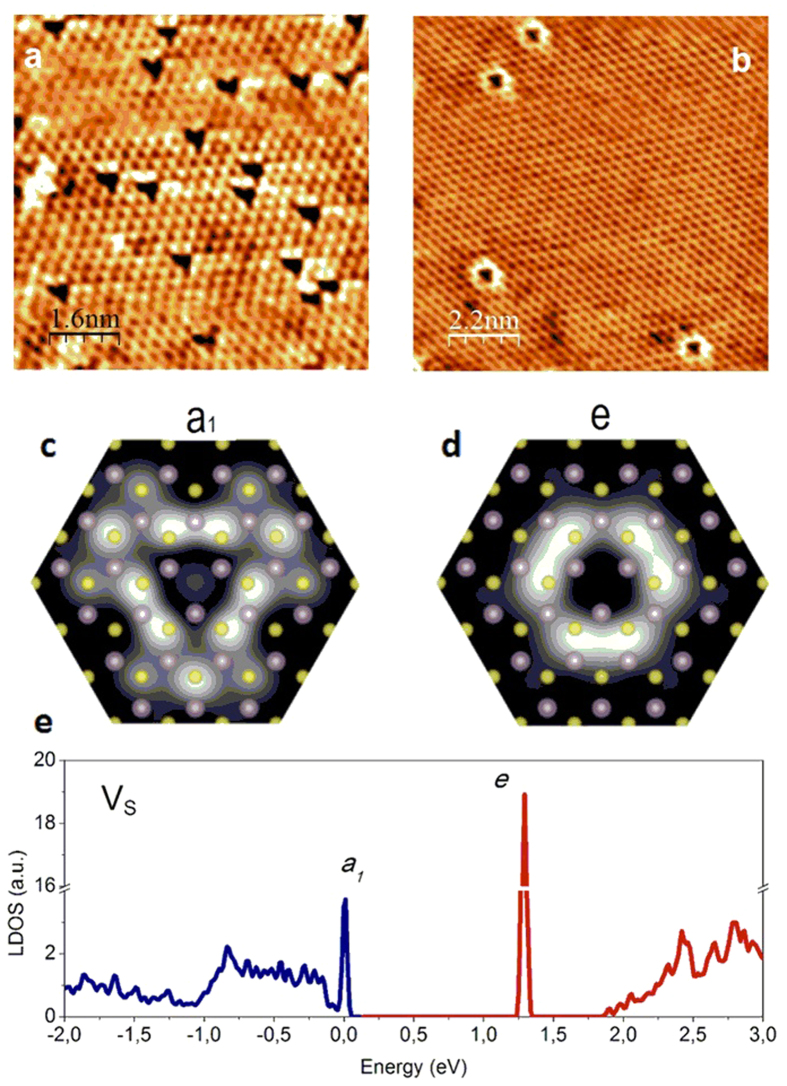
Imaging defect-induced electronic mid-gap states in MoS_2_ single layers. (**a**,**b**) Atomic resolution STM images (*V*_*b*_ = 50 mV, *I*_*t*_ = 3 nA) of single-layer MoS_2_ with triangular and circular shaped point defects, acquired under similar conditions, but at different spatial locations of the same flake. (**c,d**) Simulated STM images of the two electronic mid-gap states (*a*_*1*_ and *e*, see subfigure e) of a S atom vacancy based on DFT calculations. The sulfur and molybdenum atoms are shown by yellow and purple circles, respectively. (**e**) Local density-of-states for a neutral sulfur atom vacancy revealing the two distinct defect-induced electronic states in the gap. The occupied states are shown by blue and the empty states by red color.

Besides the frequently observed defects of triangular shape in the atomic resolution STM images (Fig. 2a) in some areas, defects of circular symmetry (Fig. 2b) have also been observed, under similar (low bias) imaging conditions, but much less frequently. The circular defects are also centered on an empty S lattice, pointing toward their S vacancy origin. It is also important to note that the triangular and the circular defects have never been observed in the same STM image, which again suggests that these defects are the distinct STM images of the S atom vacancies. To fully clarify this, a detailed theoretical understanding of the defect induced electronic states is required, as STM images contain a combination of electronic and structural information.

In order to clarify the origin of the STM images of the experimentally observed point defects we have performed density functional theory (DFT) calculations (see Supplementary Information for details). In our calculations one S atom from the top S layer is removed in the supercell geometry. After the relaxation, the defect preserves its trigonal (C_3v_) symmetry while three localized electronic defect states are generated in the band gap (Fig. 2e): a singlet *a*_*1*_ and a doublet *e* state^33^. The *a*_*1*_ level is located very close to the valence-band maximum (VBM) and the intrinsic Fermi level, while the doubly degenerate unoccupied states (*e*) are found deep inside the band gap (Fig. 2e). It is tempting to assume, that the experimentally imaged two defect types correspond to the two possible electronic states of the S vacancy defect. In order to confirm this we have calculated the local density of states of the neutral *a*_*1*_ and *e* states, near the S vacancy. Indeed the simulated STM images corresponding to the two defect states (Fig. 2c,d) display a triangular (*a*_*1*_ state) and a closely circular (the doublet *e* states) symmetry, in excellent agreement with the experimental findings. The comparison of the calculated LDOS with the STM measurements is justified since our STM images have been acquired at low bias voltages of 50 meV, which implies that we image the LDOS only in the close vicinity of the Fermi level. Consequently, the defect induced electronic states can be clearly resolved when the Fermi level of the system is in the close vicinity of one of the defect states. Since the *a*_*1*_ and *e* states (Fig. 2a,b) are located further apart in energy, this implies a considerable variation of the Fermi level as a function of sample location. The inhomogeneous spatial distribution of the Fermi level in MoS_2_ single layers is clearly confirmed by transport measurements, experimentally identifying both n- and p-type areas within the same MoS_2_ flake deposited on gold substrate^11^. Consequently, depending on location, the Fermi level can be close either to the *a*_*1*_ state (when the triangular shape is clearly observed) or the *e* state (when the circular shape is apparent), but of course not both. This explains why we do not observe the coexistence of the triangular and circular defect states. Furthermore, the clear dominance of the triangular *a*_*1*_ states observed in measurements can be understood by considering that the intrinsic position of the Fermi level (according to our DFT calculations, and tunneling spectroscopy data) is much closer to the *a*_*1*_ state. Circular *e* states only appear in STM images when the Fermi level is heavily shifted from its original position. We attribute the significant spatial variation of the Fermi level (doping) over restricted areas to the variation of the interaction strength of the MoS_2_ layer and the Au (111) substrate. We propose that although most of the interface is atomically clean, more weakly interacting areas also occurs due to contaminant intercalation, inducing a significant shift in the Fermi level position.

Tunneling spectroscopy on individual point defects is able provide quantitative data on the precise energies of the defect induced electronic states. However, at room temperature it is not possible to stabilize the STM tip above a single atomic site due to the large thermal drift. Nevertheless, we were able to perform reproducible spectroscopy measurements near the defect sites (see Fig. S2 in the SI). The spectroscopy data indeed points out that in most sample locations the Fermi level is located close to the valence band and hence the triangular *a1* state is observed most often in topography. Nevertheless, in some sample areas it also can be significantly shifted, which also enables us imaging the circular *e* state.

Line defects formed by S vacancies were found to be particularly stable from calculations^35^ and they have been experimentally observed in atomic resolution TEM investigations^20^. However, no such defects have been observed in our STM investigations suggesting that they are not native defects of the MoS_2_ single layers, but such linear agglomeration of sulfur vacancies can only occur under electron beam irradiation of the TEM measurement. The required energy of the exchange between a S atom and the neighboring S vacancy, namely the migration barrier, is calculated to be 2.3 eV at the PBE level^20^,^35^. However, it is important to note that due to this high migration barrier S vacancies are not expected to be able to diffuse at room temperature conditions. Our STM measurements directly confirm these calculations as the observed S vacancies were immobile during long scanning times, and no further vacancies have been formed during the imaging.

**Figure 3:**
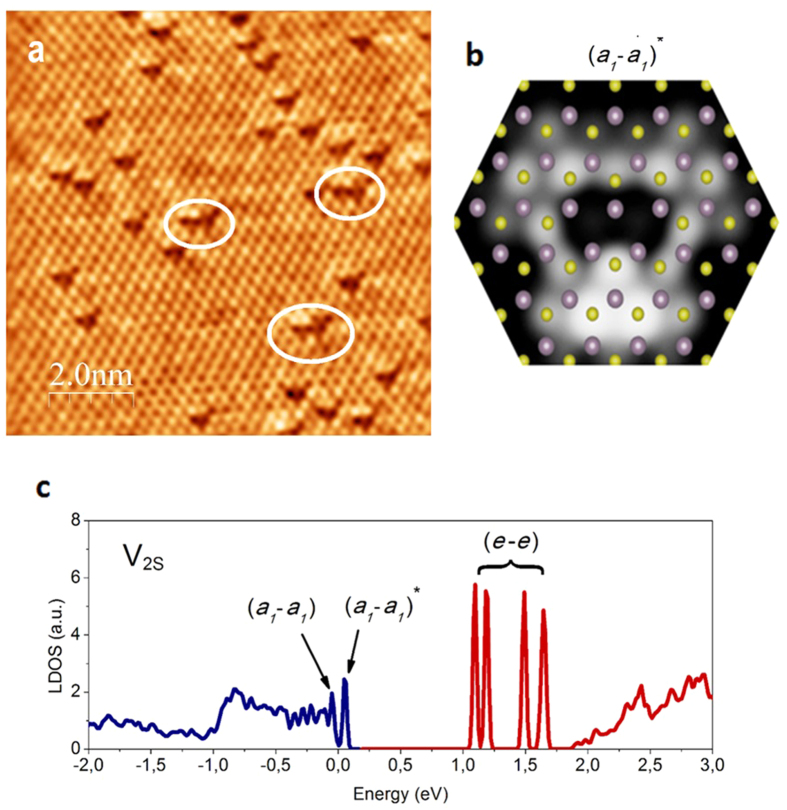
Disulfur vacancies in single-layer MoS_2_. (**a**) Atomic resolution STM images (*V*_*b*_ = 50 mV, *I*_*t*_ = 2 nA) of single-layer MoS_2_ with triangular shaped defects right next to each other forming a disulfur vacancy (V_2S_) marked by white ellipses. (**b**) Simulated STM images of the neutral disulfur vacancy (V_2S_) *a*_*1*_ state based on DFT calculations. (**c**) Density-of-states for neutral V_2S_ where the (*a*_*1*_ − *a*_*1*_) and (*e* − *e*) divacancy hybridized defects states are marked. The occupied states are shown by blue and the empty states by red color.

In the experimental STM images (Fig. 3a), besides the individual S vacancies, also pairs of triangular shape defects (marked by white circles) have been observed, which form disulfur vacancies (V_2S_’s). To confirm this observation we have also calculated the STM image of a sulfur divacancy using DFT calculations where two neighboring S atoms from the top S layer are removed. Although the atomic structure of a sulfur divacancy does not considerably differ from that of two neighboring sulfur vacancies, they have a positive binding energy (40 meV) and their electronic state is different due to hybridization effects. The *a*_*1*_ and *e* states of a single V_S_ are hybridized with those of the other neighboring V_S_ in the V_2S_. The occupied (*a*_*1*_ − *a*_*1*_) bonding and (*a*_*1*_ − *a*_*1*_)* antibonding levels are generated near the VBM, and the four (*e* − *e*) hybridized states are induced in the band gap. The lowest (*a*_*1*_ − *a*_*1*_) bonding level is found to be located inside the valence band, as indicated in Fig. 3c, and thus largely hybridized with the host states, while the second lowest (*a*_*1*_ − *a*_*1*_)^*^ anti-bonding level is just above the VBM inside the bandgap and thus clearly distinguished from the host states. This (*a*_*1*_ − *a*_*1*_)^*^ state is closest to the Fermi level therefore it is expected to be predominantly observed in the STM images. The good agreement of the simulated STM image (Fig. 3b) of the (*a*_*1*_ − *a*_*1*_)^*^ state with the experimental observations (Fig. 3a) confirms this expectation and the presence of native disulfur vacancies (V_2S_) in our single-layer MoS_2_ sample. However, their concentration was experimentally found to be much lower as compared to single S atom vacancies. Previous calculations predicted that the migration barrier of the V_2S_ can be significantly lower (0.8 eV) compared to V_S_ (2.3 eV) 35. This lower barrier corresponds to the process where the V_2S_ rotates by 60° degrees instead of forming two individual V_S_’s where the missing two S atoms are in the next-nearest-neighbor configuration. The fact that in our STM measurements the position of V_2S_ defect has not changed during repeated scanning, namely no rotation has been observed, further strengthens our previous observation that the native point defects of MoS_2_ single layers are immobile at room temperature and STM does not perturb the intrinsic defect structure of MoS_2_ single layers.

## Conclusions

In conclusion, we were able to fully resolve individual atomic scale defects of exfoliated MoS_2_ single layers, by STM imaging at energies within the band gap. A high native point defect concentration of order of 10^13^ cm^−2^ could be directly imaged. The dominant defects have been identified as single sulfur atom vacancies, as also suggested by their low formation energies found in DFT calculations. Beside single S vacancies a low concentration of S divacancies has also been identified. Our STM measurements clearly evidence that S atom vacancies are immobile at room temperature, again in good agreement with the relatively high migration energy barrier predicted by theory. Combined with DFT calculations, our STM investigations were able to identify two midgap electronic states localized on the defects, and appearing as features of trigonal and circular symmetry. These states can be identified as the *a*_*1*_ and *e* electronic defect states predicted by *ab inito* calculations. The experimental findings reported here, concerning the defect-induced electronic states reveal the unavoidable role of defects in the operation of realistic electronic devices based on 2D crystals of molybdenum disulfide. Besides electronic device application, the native defects of MoS_2_ are expected to play a technologically relevant role in several applications, such as optoelectronics and catalysis.

## Additional Information

**How to cite this article**: Vancsó, P. *et al*. The intrinsic defect structure of exfoliated MoS_2_ single layers revealed by Scanning Tunneling Microscopy. *Sci. Rep.* **6**, 29726; doi: 10.1038/srep29726 (2016).

## Supplementary Material

Supplementary Information
