# Supplementary material for: The intrinsic defect structure of exfoliated MoS2 single layers revealed by Scanning Tunneling Microscopy
Source: Sci Rep. 2016 Jul 22;6:29726. doi: 10.1038/srep29726 (PMC4957227; doi:10.1038/srep29726)
Supplement: Supplementary Information [file srep29726-s1.pdf]

Supplementary Information

The intrinsic defect structure of exfoliated MoS_2 single layers revealed by Scanning Tunneling Microscopy

Péter Vancsó, Gábor Zsolt Magda*, János Pető, J.Y. Noh, Yong-Sung Kim, Chanyong Hwang, László P. Biró, and Levente Tapasztó*

I. Sample preparation

The MoS_2 single layers have been exfoliated onto Au (111) substrates using a novel mechanical exfoliation technique developed by us, and described elsewhere [1], yielding single layers of several hundreds of microns lateral size. To ensure the high structural quality of the investigated samples we used synthetic bulk MoS_2 crystals (2DSemiconductors), which we found of higher quality than naturally occurring crystals investigated by us. The exfoliation process involves only a mild sonication step of a few seconds, which is not expected to induce any additional defect sites as samples sonicated for longer times did not display a higher defect concentration. The single layer nature of such flakes has been confirmed by a combination of optical microscopy, Raman spectroscopy as well as STM measurements of step height relative to the gold substrate near the MoS_2 flake edges.

II. Scanning Tunneling Microscopy and Spectroscopy characterization

STM investigations have been performed on a Nanoscope E instrument, at room temperature, under ambient and in pure N_2 atmosphere, using an atmospheric hood, in order to protect the samples from oxidation during scanning with higher bias voltages (>1V). Landing the STM tip on a single layer MoS_2 flake has been achieved by guiding the tip under an optical microscope. This had been made possible by the large (>100 microns) lateral size of the exfoliated single layer flakes. Typical imaging parameters for atomic resolution were 5 – 50 mV, and 1 – 3 nA. To clearly resolve the atomic structure of point defects, ideal tip conditions and the fine tuning of the imaging parameters was needed. In contrast, to the apparent resolution of the hexagonal lattice of the top S atoms, the high quality images clearly resolving individual point defects could not be easily achieved. Typical STM images of point defect in MoS_2 single layers under non-ideal imaging conditions are shown in Fig S1a.

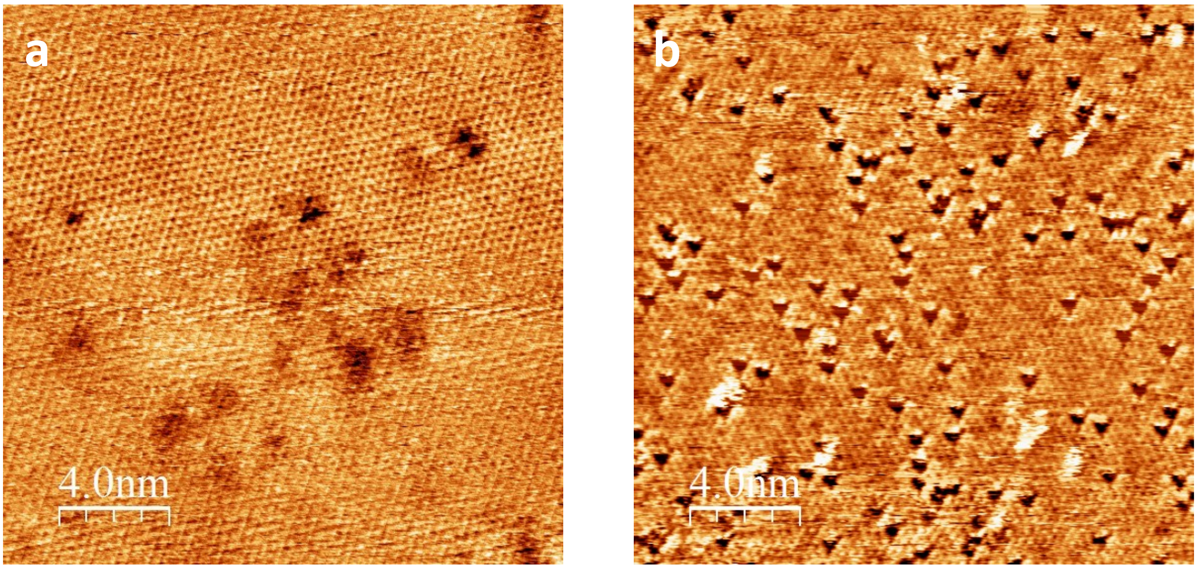

Fig. S1. *STM image of point defects in MoS_2 single layers acquired under non-ideal imaging conditions (10 mV, 2nA), when the Fermi level is located away from the defect states (a) and under ideal imaging conditions (50mV, 2 nA), in a different sample area (b).*

Reliable tunneling spectroscopy over a single point defect could not be achieved due to the thermal drift at the room temperature. However, reproducible tunneling spectra could be acquired near the defect sites. A typical spectrum is shown in Fig S2.a.

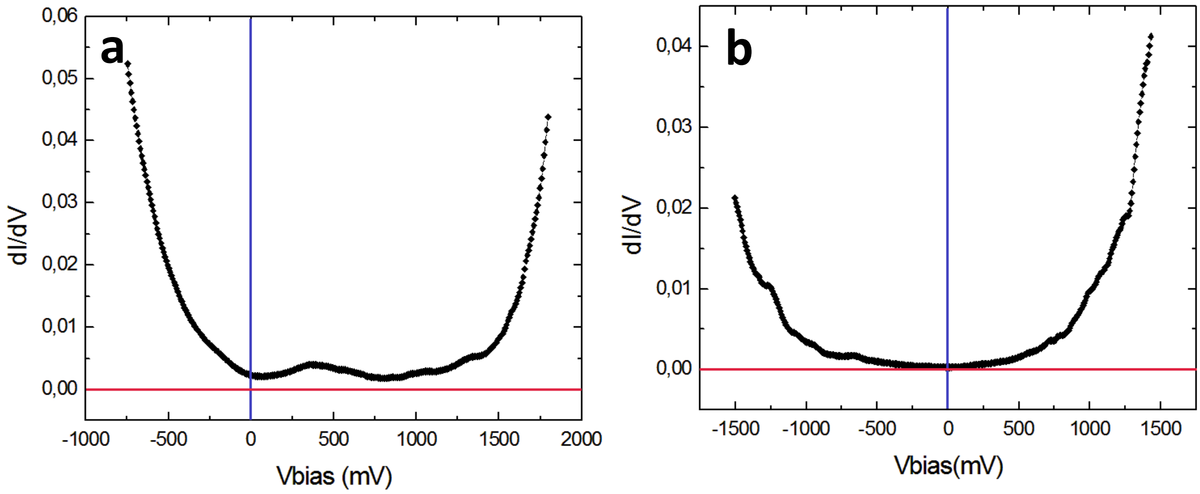

Fig. S2. *Tunneling spectra acquired on MoS_2 single layer deposited on Au (111) substrate.*

The tunneling spectra display a band gap of about 1.8 eV in excellent agreement with previous results. However, the density of states within the gap has a small but finite value, which we attribute to the presence of the Au substrate. It is also apparent that the Fermi level (vertical blue line) is close to the valence band maximum While the spectrum in Fig S2 a, has been predominantly observed, in a few areas spectra with significantly shifted Fermi level position have also been observed (Fig. S2 b), evidencing the spatial inhomogeneity of the doping, which we attribute to the variation of the interaction strength between the MoS_2 layer and the Au (111) substrate, due to impurity intercalation in restricted areas.

III. Density Functional Calculations

The DFT calculations were performed within the framework of local density approximation (LDA) using the Vienna *ab initio* simulation package (VASP) [2,3]. Projector augmented wave (PAW) pseudo-potentials [4,5] were used and the kinetic energy cut-off for the plane wave expansion was 350 eV. We applied 8×8 surface unit cell (192 host atoms) where the in-plane lattice constant (distance between neighbor S atoms) was 3.124 Å from our previous LDA calculations [6] and the vacuum thickness was 15 Å. The atomic structures were relaxed until the Hellmann-Feynman forces are less than 0.02 eV/Å. The Γ- point was used for the Brillouin-zone summation. STM images were simulated with the simple Tersoff-Hamann approximation [7] using the calculated local density of states.

IV. Sulfur Divacancies

The atomic structures of V_S and V_{2S} are compared in Fig. S3 a and b. With the V_{2S}, the surrounding Mo atoms get closer to each other, as shown by the Mo-Mo bonding (red lines) in Fig. S3b. The defect level hybridization due to the binding of two V_S's is schematically drawn in Fig. S3c. The bonding and anti-bonding states of (a_1-a_1) and (a_1-a_1)* are generated by the hybridization of the singlet a_1 states, while four (e-e) states are generated by the double e state hybridization in a divacancy. The (a_1-a_1) bonding state is very close to the host VB and coupled with the host VB sates. The (a_1-a_1)* antibonding state is found to be characteristically observed in STM for the V_{2S} defect.

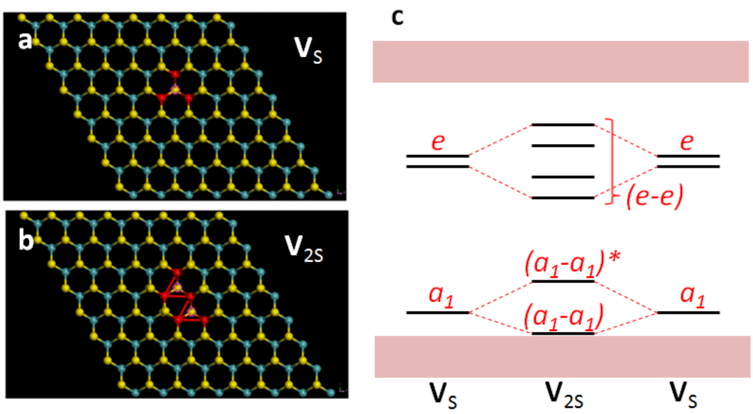

Figure S3. *Atomic structure and schematic bandstructure of disulfur vacancy (V_{2S}).*

References

[1] G. Zs. Magda, J. Pető, G. Dobrik, C. Hwang, L. P. Biró, L. Tapasztó, Exfoliation of large-area transition metal chalcogenide single layers. Sci Rep. 2015; 5:14714.

[2] G. Kresse, J. Hafner, Ab initio molecular-dynamics simulation of the liquid-metal amorphous-semiconductor transition in germanium. Phys. Rev. B 1994; 49(20):14251–14269.

[3] G. Kresse, J. Furthmüller, Efficient iterative schemes for ab initio total-energy calculations using a plane-wave basis set. Phys. Rev. B 1996; 54(16):11169–11186.

[4] G. Kresse, D. Joubert, From ultrasoft pseudopotentials to the projector augmented-wave method. Phys. Rev. B 1999; 59(3):1758–1775.

[5] P.E. Blöchl, Projector augmented-wave method. Phys. Rev. B 1994; 50(24):17953–17979.

[6] J.-Y. Noh, H. Kim, Y.-S. Kim, Stability and electronic structures of native defects in single-layer MoS2. Phys. Rev. B 2014; 89:205417.

[7] J. Tersoff, D.R. Hamann, Theory and Application for the Scanning Tunneling Microscope. Phys. Rev. Lett. 1983; 50(25):1998–2001.
